# The impact of intratumor heterogeneity on outcome and resistance to therapy in primary breast cancer: a systematic review

**DOI:** 10.1007/s12032-026-03426-z

**Published:** 2026-10-03

**Authors:** Aria Dianati, Andre Dekker, Anke Wesselius, Leonard Wee

**Affiliations:** 1 Department of Radiation Oncology (Maastro), GROW Research Institute for Oncology and Reproduction, Maastricht University Medical Centre, Maastricht, The Netherlands; 2 Department of Epidemiology, NUTRIM School of Nutrition and Translational Research in Metabolism, , Maastricht University, Maastricht, The Netherlands

**Keywords:** ITH, Breast cancer, Chemotherapy, Treatment resistance

## Abstract

Intratumor heterogeneity (ITH), the coexistence of diverse subclones within a single tumor, may pose a major challenge to effective chemotherapy in breast cancer. This cellular diversity contributes to treatment failure, residual disease, and early relapse by enabling tumor adaptation and therapeutic resistance. Although increasingly recognized as a critical feature of breast cancer biology, it remains under-investigated and has not yet been integrated into routine clinical care. This review synthesizes current evidence on the role of ITH in chemotherapy resistance in primary breast cancer. Following PRISMA guidelines, we searched MEDLINE via PubMed, Web of Science, and Scopus through June 2026. Twelve studies met the inclusion criteria. Data were extracted on tumor subtypes, study design, ITH assessment modality (genomic, transcriptomic, radiomic, or immunologic), sampling method, treatment type, and outcomes. Among the 12 included studies, covering triple-negative, HER2-positive, and mixed breast cancer cohorts, high or increasing ITH, assessed using molecular or imaging-based methods, was associated with reduced sensitivity to chemotherapy. Proposed mechanisms underlying resistance included clonal selection, transcriptional plasticity, immune exclusion, and phenotypic drift. Radiomic and MRI-derived features may provide additional predictive value beyond molecular classifiers, and integrated radiomic-genomic models outperformed either modality alone in identifying patients with poor treatment response. In conclusion, current evidence suggests that ITH may be associated with chemotherapy resistance in primary breast cancer through several biological mechanisms. Integrating radiomic and imaging-based assessments with molecular profiling has the potential to improve the prediction of treatment response and facilitate monitoring of changes in tumor heterogeneity during therapy, although further prospective validation is needed before routine clinical implementation.

Protocol Registration Number at PROSPERO: CRD42024533885

## Highlights

Higher or increasing ITH is associated with chemotherapy resistance in primary breast cancer.

MRI-based ITH metrics seems to be associated with treatment response and recurrence risk.

Pre-existing resistant subclones and transcriptional reprogramming may contribute to chemotherapy resistance.

Immune exclusion and MALAT1 upregulation may contribute to chemoresistance in TNBC.

## Introduction

According to the World Health Organization (WHO) breast cancer remains the most commonly diagnosed cancer among women worldwide, with over 2.3 million new cases each year [1]. Despite significant advances in systemic therapies such as neoadjuvant chemotherapy (NAC), targeted therapy, and immunotherapy treatment resistance continues to limit clinical outcomes, particularly in aggressive subtypes like triple-negative breast cancer (TNBC) and HER2-positive disease [2, 3]. Therapeutic resistance, whether intrinsic or acquired, is a major challenge across oncology [4]. While breast cancer treatment has evolved considerably, intratumor heterogeneity (ITH) remains an under-recognized contributor to suboptimal response and disease progression [5].

Chemotherapy resistance arises from both tumor cell–intrinsic factors (e.g., somatic mutations, epigenetic changes, transcriptional plasticity) and tumor microenvironmental influences, such as stromal remodeling, immune evasion, and drug efflux mechanisms [6, 7]. Recent research has made substantial progress in identifying predictive biomarkers and dissecting cellular programs that underlie resistance [8]. Gene expression assays, for instance, have improved patient stratification. In estrogen receptor-positive (ER+) breast cancer, the 21-gene Recurrence Score (Oncotype DX) helps identify patients unlikely to benefit from chemotherapy [9]. Albain et al. demonstrated in a randomized trial that postmenopausal women with node-positive, ER+ breast cancer and a low Recurrence Score (< 18) had no improvement in survival from adding anthracycline-based chemotherapy to endocrine therapy [10]. In other words, a low genomic-risk tumor behaved as intrinsically chemo resistant, whereas those with high scores benefited significantly from chemotherapy. This highlighted how genomic profiling can predict chemo non-responsiveness in certain subsets [11, 12].

A growing body of evidence implicates ITH as a central driver of chemotherapy resistance and recurrence [5]. ITH refers to the coexistence of genetically, transcriptionally, epigenetically, and phenotypically distinct cell populations within a single tumor [13, 14]. Accordingly, genetic ITH includes somatic mutations, copy number alterations, and chromosomal instability. Also, transcriptomic ITH involves the dynamic expression of resistance-associated pathways (e.g., epithelial-mesenchymal transition [EMT], DNA repair) [15]. Spatial and radiologic heterogeneity, measurable through advanced imaging techniques like MRI and radiomics, reflect differences in tumor architecture, vascularization, and perfusion and immune ITH denotes regional variability in immune infiltration and checkpoint expression [16]. These various dimensions of ITH contribute to incomplete tumor regression, residual disease, and early recurrence following chemotherapy [16, 17].

Despite increasing evidence that ITH contributes to chemotherapy resistance and relapse, its role in breast cancer is still not fully understood or applied in practice. Progress has been limited by the absence of standardized ways to measure ITH across different modalities, such as sequencing, radiomics, spatial imaging, and immunogenomics, and by treatment decisions that remain largely based on bulk tumor features, which overlook the tumor’s internal diversity. This systematic review brings together current evidence on how ITH contributes to chemotherapy resistance in primary breast cancer, compares approaches used to capture heterogeneity, identifies outcome markers linked to high ITH, and highlights opportunities to integrate molecular and imaging data into future treatment strategies aimed at improving response prediction and patient stratification.

## Methods

### Study design

This review was conducted following the PRISMA (Preferred Reporting Items for Systematic Reviews) guidelines [18], under a registered protocol at the international PROSPERO (Registration Number: CRD4202453 3885).

### Eligibility criteria (PICOS)

The PubMed/Medline, Web of Science, and Scopus databases were searched for published studies up to June 2026, and the search was restricted to peer-reviewed articles published in English. Medical Subject Headings (MeSH) and non-MeSH terms, including keywords and their synonyms related to ITH, breast cancer, chemotherapy, and treatment resistance, were used in combination with Boolean operators (AND/OR) to optimize the search strategy across all databases. Studies were included if they met the following criteria: original research articles, either prospective or retrospective in design, that focused on primary breast cancer; studies that explored ITH through genomic, transcriptomic, radiomic, or immunologic approaches; and studies that assessed chemotherapy resistance, therapeutic response, or clinical outcomes following chemotherapy. Additionally, eligible studies were required to provide sufficient data for extraction, including details on sample size, methodologies, and reported outcomes. Exclusion criteria comprised case reports and case series, reviews, and editorials, as well as studies exclusively examining metastatic breast cancer or those that did not evaluate ITH in relation to chemotherapy response or clinical outcomes.

### Data extraction process

Two reviewers independently reviewed the selected articles and extracted key variables into structured tables for consistent comparison and analysis. These variables included study identification details such as author, year, and title, as well as the study design. Also, sample characteristics were recorded, including the total number of patients and subtypes of breast cancer, such as TNBC, HER2-positive, or mixed populations. The modality used to assess ITH was examined, covering genomic, radiomic, scRNA-seq, or immune-based approaches.

Each included study was evaluated for quality and risk of bias using standardized assessment tools appropriate to its design. Cohort studies were assessed using a modified version of the Newcastle-Ottawa Scale (NOS) [19], while clinical trials were evaluated using the Cochrane Risk of Bias tool [20]. The assessment criteria focused on several key aspects, including the clarity and specificity of the outcome definitions, the appropriateness and reliability of the techniques used to measure ITH, the adequacy of the follow-up duration to capture relevant outcomes, and the extent to which studies controlled for confounding variables and demonstrated statistical rigor. Although studies identified as having a high risk of bias were clearly noted, they were not excluded from the review due to their exploratory scope and the limited availability of data in this emerging field.

### Data synthesis

A synthesis approach was employed to accommodate the clinical and methodological heterogeneity observed across the included studies. Findings were organized thematically based on tumor subtypes such as TNBC, HER2-positive, or mixed populations as well as the modality used to assess ITH, including genomic, transcriptomic, radiomic, and immune-based methods. Additionally, studies were grouped by the resistance mechanisms identified, such as adaptive selection, transcriptional reprogramming, or immune exclusion.

### Ethical considerations

This review relied on publicly available literature and data extracted directly from the publications. No human participants were involved in the analysis, and therefore, ethics approval was not required.

## Results

### Study selection and characteristics

**Fig. 1:**
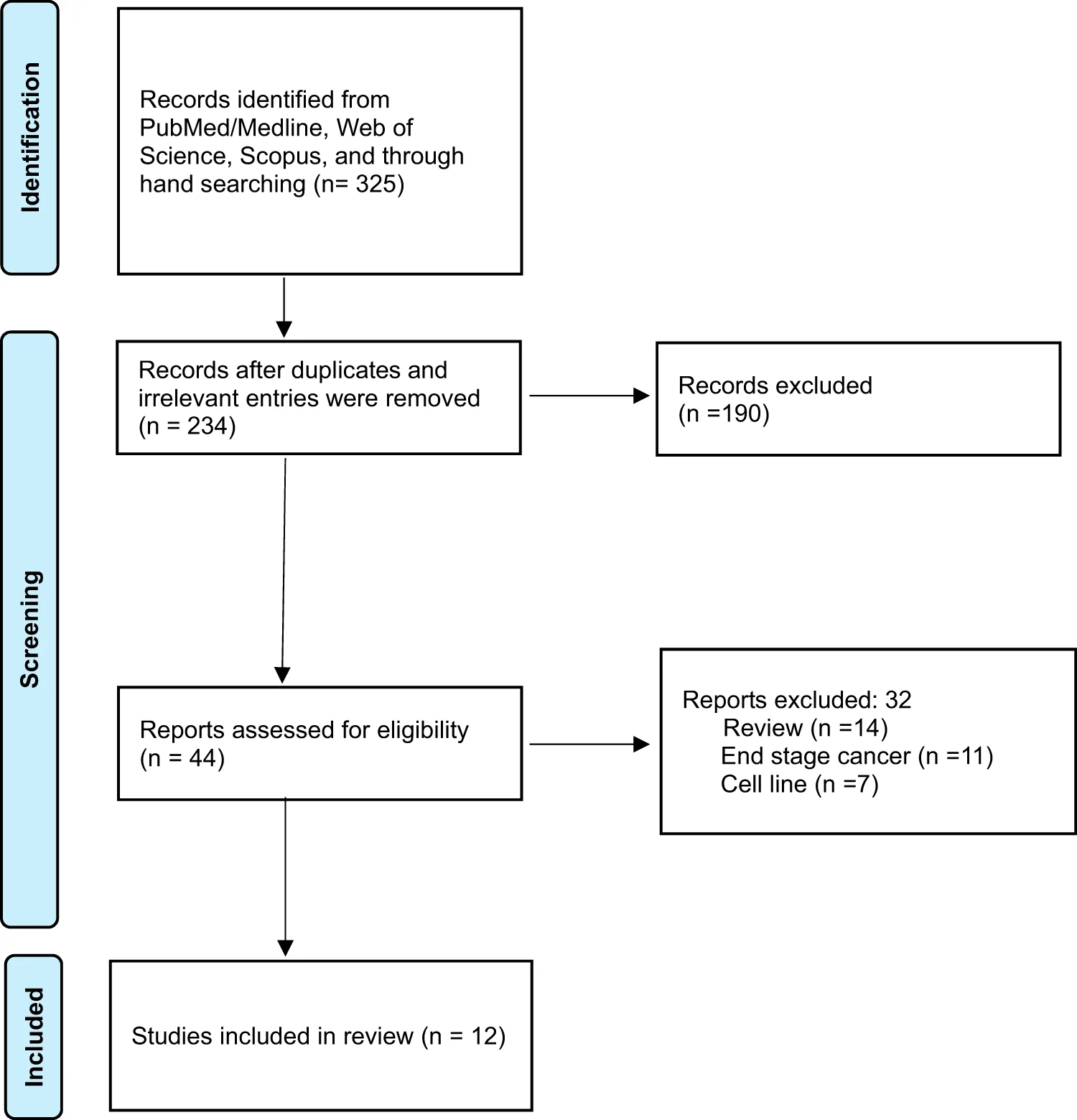
PRISMA 2020 flow diagram for systematic review

A total of 325 potentially relevant articles were identified through the database search. After removing 91 duplicate records, 234 articles remained for title and abstract screening. Of these, 190 were excluded for not meeting the inclusion criteria. The full texts of the remaining 44 articles were reviewed in detail. Subsequently, 32 articles were excluded for the following reasons: review articles (*n* = 14), studies involving end-stage cancer or non-breast cancer types (*n* = 11), and studies not evaluating patient data (*n* = 7). Ultimately, 12 studies met all eligibility criteria and were included in the current review. The selection process is illustrated in the PRISMA flow diagram (Fig. 1).

All selected studies focused on primary breast cancer and examined TNBC [21–24], HER2-positive [25–27], HR [28], and mixed subtypes of breast cancer [29–32]. Each study explicitly investigated ITH using an analytical method (genomic, transcriptomic, radiomic, and immunologic). Notably, the included studies examined treatment response or resistance in the context of chemotherapy. Sample sizes varied in the included studies, ranging from 6 to 1589 patients. The methodologies used across studies included single-cell RNA sequencing, MRI-based radiomics, long non-coding RNA (lncRNA) profiling, and spatial immune assays.

A detailed summary of these key study characteristics is provided in Table 1.

**Table 1:** Summary of key characteristics of included studies

Author	Year	Study Design	Sample Size	Cancer Type	Hormone Receptor Status	Outcome Measure
Zhu et al. [29]	2025	Retrospective cohort	217 patients	Primary breast cancer	Mixed (HER2+, HR+)	pCR
Hatano et al. [26]	2025	Retrospective study	97 patients	Primary breast cancer	HER2+	pCR
Dai et al. [27]	2025	Retrospective study	205 patients	Primary breast cancer	HER2+	pCR
Huang et al. [28]	2025	Retrospective study	1448 patients	Primary breast cancer	HR + and HR -	pCR, RFS
Tarantino et al. [25]	2024	Prospective cohort	512 patients	Primary breast cancer	HER2+	iDFS, RFI, OS, BCSS
Gandhi et al. [21]	2024	Clinical trial	9 patients	Primary breast cancer	TNBC	pCR, RFS, OS
Lusby et al. [22]	2024	Translational study	7 patients	Primary breast cancer	TNBC	pCR
Shi et al. [32]	2023	Retrospective study	1589 patients	Primary breast cancer	Mixed (HR+, HER-2+, TNBC)	pCR
Chitalia et al. [30]	2023	Prospective cohort	192 patients	Primary breast cancer	Mixed (HR+, HER-2+, TNBC)	pCR
Shaath et al. [23]	2021	Experimental study	6 patients	Primary breast cancer	TNBC	lncRNA MALAT1 as a regulator of TNBC resistance to NAC
Kim et al. [24]	2018	Prospective cohort	20 patients	Primary breast cancer	TNBC	Clonal extinction vs. persistence post-chemotherapy
Almendro et al. [31]	2014	Prospective cohort	47 patients	Primary breast cancer	Mixed (luminal A, Luminal B, HER2+, TNBC)	pCR

pCR= pathologic Complete Response, IDFS= invasive Disease-Free Survival, RFI= Recurrence-Free Interval, OS= Overall Survival, RFS = Recurrence-Free Survival, BCSS= Breast Cancer–Specific Survival.

In the included studies, diverse NAC regimens were paired with radiomic, genomic, transcriptomic, immune, and single-cell methods to assess ITH. Genomic and transcriptomic analyses identified specific molecular drivers of resistance, including HER2 heterogeneity and lncRNA MALAT1, and immune profiling revealed enhanced CD8 + T-cell infiltration in responders. Treatment regimens, ITH assessment methods, and key ITH-related findings in the included studies are summarized in Table 2.

**Table 2:** Treatment regimen, ITH assessment methods, and key ITH-related findings in included studies

Author	Treatment Type	Chemotherapy Regimen	ITH Type	Assessment Techniques	Key ITH Findings
Zhu et al. [29]	NAC	Paclitaxel-and-platinum-based NAC	Radiographic ITH from dynamic contrast-enhanced MRI	MRI-based heterogeneity quantification	Lower pre-treatment MRI heterogeneity was significantly associated with achieving pCR. Rising ITH during NAC independently predicted worse overall survival
Hatano et al [26]	NAC	Anti-HER2 NAC	Genetic HER2 positive heterogeneity	HER2 FISH histogram analysis with Gaussian mixture model (GMM)	High HER2 heterogeneity was independently associated with significantly lower pCR rates and anti-HER2 resistance
Dai et al. [27]	NAC	Trastuzumab-containing anti-HER2 NAC regimens	HER2 genetic ITH	HER2 fluorescence in situ hybridization (FISH), HER2 immunohistochemistry (IHC), pathologic evaluation	HER2 ITH was associated with reduced response to anti-HER2 neoadjuvant therapy
Tarantino et al. [25]	AC	Paclitaxel and Trastuzumab	Genomic HER2 positive heterogeneity	HER2DX assay - multi-omics analysis (genomics + proteomics)	HER2DX-defined low-risk tumors had significantly better relapse-free survival. HER2 heterogeneity linked to recurrence risk independently of treatment type
Huang et al. [28]	NAC	Chemotherapy ± HER2-targeted therapy	MRI-based radiomic/spatial ITH	Dynamic contrast-enhanced MRI (DCE-MRI), sliding-window radiomics, multiscale ITH scoring	MRI-based ITH score independently predicted pCR and recurrence-free survival
Gandhi et al. [21]	NAC	Paclitaxel, doxorubicin/ cyclophosphamide	Immune-based ITH	Flow cytometry, RT-qPCR, MICSSS	Increased intratumor CD8⁺ T-cell infiltration and a higher CD8⁺/FoxP3⁺ ratio, chemokine upregulation (CCL5, CXCL12) promoting immune cell recruitment, closer CD8⁺ T-cell–tumor cell interactions, reduced circulating CD8⁺ T cells
Lusby et al. [22]	NAC	Anthracycline + Taxane	Transcriptomic and epigenomic	Quantitative multiplex immunofluorescence, and RNA-based gene pathway signatures	Pre-existing basal epithelial cell subpopulations with distinct gene expression and regulatory networks. These resistant cells are controlled by subtype-specific super-enhancers and core transcription factors, especially TFAP2C and SP1
Shi et al. [32]	NAC	Taxane-based, alkylator-based, and anthracycline-based chemotherapy	MRI-derived radiomic/ecological ITH	Intratumor subregion segmentation; ecological diversity feature extraction; conventional radiomics; machine-learning	MRI-based ITH index independently predicted pathologic complete response (pCR). Higher ecological heterogeneity significantly improved predictive modeling performance when combined with clinicopathologic and radiomics variables
Chitalia et al. [30]	NAC	Anthracycline- and taxane-based standard NAC	MRI-based radiomic, transcriptomic	Pre- and early-treatment DCE-MRI for voxel-wise kinetic mapping - Radiomic features extracted to compute heterogeneity change, MammaPrint	Two principal imaging phenotypes of ITH were significantly associated with recurrence-free survival, and integrating imaging with genomic data improved predictive performance.
Shaath et al. [23]	NAC	Paclitaxel and Doxorubicin	Transcriptomic	ICGS, CRISPR/Cas9, RT-qPCR, CFU assay, Sanger sequencing	lncRNA MALAT1 is markedly upregulated in resistant tumors, discriminates resistant from sensitive phenotypes, and functionally promotes chemoresistance, as its knockout enhances sensitivity to paclitaxel and doxorubicin and alters lncRNA–mRNA regulatory networks
Kim et al. [24]	NAC	Epirubicin and docetaxel	Genetic (mutation, copy number alterations), and transcriptomic	Whole-exome sequencing (bulk), single-nucleus sequencing (DNA), single-nucleus RNA sequencing, computational clonal inference tools (PyClone2, CITUP)	Resistance in TNBC stems from pre-existing adaptive clones, followed by therapy-induced transcriptional reprogramming without added mutation burden. Common resistance traits include EMT, AKT1 activation, hypoxia, angiogenesis, and ECM degradation.
Almendro et al. [31]	NAC	Anthracyclines	Genetic and phenotypic	Immunofluorescence, Ki67 proliferation assay, Shannon and Simpson diversity indices, spatial topology mapping	Phenotypic heterogeneity is highly dynamic during chemotherapy, with treatment-induced shifts in tumor cell populations (including increased CD44⁻/CD24⁺ and CD44⁻CD24⁻ cells) and, in some cases, greater spatial heterogeneity, while pre-treatment genetic diversity, particularly 8q24 copy number variation, correlates with therapeutic response

MICSSS= Multiplexed Immunohistochemistry, ICGS= Iterative Clustering and Guide-gene Selection, CFU assay= Colony Forming Unit assay. CD8⁺= Cluster of Differentiation 8–positive (cytotoxic T lymphocytes), FoxP3 = Fork head Box P3 (regulatory T-cell transcription factor), TFAP2C= Transcription Factor AP-2 Gamma, SP1 = Specificity Protein 1, MALAT1: Metastasis-Associated Lung Adenocarcinoma Transcript 1, AKT1 = AKT Serine/Threonine Kinase, ECM = Extracellular Matrix, CD44⁻= Cluster of Differentiation 44–negative, CD24⁺= Cluster of Differentiation 24–positive, 8q24 CNV= Copy Number Variation at chromosomal region 8q24.

### Narrative evaluation of studies

Zhu et al. [29] examined MRI-based measures of ITH as predictors of treatment response. The approach provided insights and improved predictive accuracy, yet its retrospective, single-center design and reliance on manual image processing limit how broadly the findings can be applied. Even so, the integration of imaging biomarkers with clinical features suggests potential value for improving response stratification.

Hatano et al. [26] found that HER2- ITH is associated with resistance to anti-HER2 neoadjuvant therapy and can be assessed using routine pathology data. Despite strengths such as objective image-based analysis, the retrospective single-center design, small high-heterogeneity subgroup, and limited molecular characterization were important limitations. These findings support HER2 heterogeneity as a potential predictive biomarker in HER2-positive breast cancer.

Tarantino et al. [25] reported five-year outcomes of adjuvant T-DM1 in stage I HER2-positive breast cancer, showing excellent survival rates alongside genomic analyses that support risk stratification. While the phase II design, modest comparator arm, and relatively small correlative subgroups restrict the strength of the conclusions, the durability of benefit and the use of biomarker-guided strategies highlight the trial’s importance for precision therapy.

Dai et al. [27] studied HER2 ITH in HER2-positive breast cancer and found that tumors with HER2- ITH, particularly those with low-level HER2 amplification, showed poorer response to neoadjuvant therapy and greater treatment resistance. Strengths included a large cohort and standardized HER2 testing, while the retrospective single-center design and missing biopsy material limited heterogeneity assessment. These findings suggest HER2- ITH may be a useful predictor of treatment response.

Huang et al. [28] developed an MRI-based ITH nomogram to predict response to NAC and RFS in breast cancer. Strengths included its multicenter design, external validation, and integration of imaging and genomic data, while the retrospective design and small survival and genomics cohorts limited generalizability. The findings support MRI-based heterogeneity assessment as a promising noninvasive biomarker for treatment response and prognosis.

Gandhi et al. [21] tested a chemokine-modulatory regimen with NAC in TNBC. The study showed encouraging safety and immune-modulating effects, with a pCR rate of 55%. Its small, single-center phase I design and short follow-up, however, limit the clinical weight of the findings. Still, the incorporation of immune profiling and the absence of major immune-related toxicities support further evaluation in larger randomized studies.

Lusby et al. [22] mapped the regulatory circuitry underlying chemoresistance in TNBC, identifying distinct cellular subpopulations, transcription factor networks, and a 20-gene signature predictive of response. While the use of retrospective datasets and a modest single-cell cohort limit the reach of the conclusions, the discovery of super-enhancer-driven programs and clinically relevant biomarkers offers potentially relevant translational insights that warrant prospective confirmation.

Shi et al. [32] used multicenter cohorts with external validation and combined MRI-derived ecological diversity features with radiomic analysis to predict pCR. The MRI-based ITH index improved prediction performance and showed strong results across validation cohorts. Strengths included the large multicenter design and integration of ecological diversity concepts into imaging analysis. Limitations included reliance on manual tumor segmentation, and possible variability in MRI acquisition protocols between centers, which may affect reproducibility and generalizability.

Chitalia et al. [30] combined radiomic features with molecular profiling to predict RFS after NAC. The multimodal model improved prognostic accuracy, though its retrospective design, dependence on trial data archived, and variability in imaging quality restrict generalizability. Nevertheless, the study demonstrates the value of linking imaging and genomics and supports prospective validation of such approaches.

Shaath et al. [23] profiled the lncRNA landscape at single-cell resolution and highlighted MALAT1 as a driver of chemoresistance. Functional CRISPR experiments reinforced this mechanistic link, showing effects on drug sensitivity and regulatory networks. Although the small patient cohort and reliance on in vitro model narrow applicability, the findings position lncRNA regulation as a compelling therapeutic avenue and a potential biomarker of response.

Kim et al. [24] used single-cell DNA and RNA sequencing to follow clonal dynamics in TNBC during chemotherapy, distinguishing cases of clonal extinction from persistence. The study provided important evolutionary insight into both adaptive selection and transcriptional reprogramming, though its small sample size and possible sampling bias limit broader interpretation. Even so, the integration of genomic and transcriptomic data offers a powerful framework for understanding resistance.

Almendro et al. [31] studied ITH before and after NAC, combining in situ analyses with computational modeling. They found that genetic diversity remained stable, while phenotypic composition shifted under treatment pressure. Although the focus on a limited set of loci and surface markers constrains generalizability, the use of spatial mapping and modeling added significant strength to the study.

### Synthesis of ITH insights

As summarized in Table 3, and derived from primary studies, diverse modalities for assessing ITH including imaging, genomic profiling, immune microenvironment analysis, and transcriptomics consistently suggested an association between ITH and chemotherapy resistance. First, elevated pre-treatment heterogeneity (e.g., genomic, phenotypic) was generally predictive of incomplete or poor response to NAC, supporting the use of ITH measures for risk stratification before treatment. Second, persistence or increase in ITH after chemotherapy, as detected through imaging or integrated radiomic–transcriptomic analyses, reflected tumor evolution under drug selection and the emergence of resistant clones. Third, temporal changes in ITH during therapy (delta-ITH), particularly increases captured via MRI, offered a promising non-invasive early indicator of poor outcomes. Lastly, immune-related heterogeneity emerged as a potentially relevant therapeutic dimension of ITH, with studies showing that targeted interventions such as Cytokine-Kinase Mediated (CKM) immune priming can remodel the tumor microenvironment, enhance cytotoxic T cell infiltration, and improve treatment efficacy.

**Table 3:** Key Findings by Heterogeneity Type and Clinical Implications

Study	ITH Aspect	Sampling Context	Role in Resistance	Therapeutic Implication
Zhu et al. [29]	Imaging-based (MRI), temporal and spatial grayscale variation	Whole-tumor MRI	Rising ITH associated with increased recurrence risk and lower pCR; suggests resistance evolution during treatment	Rising ITH may help identify high-risk patients for intensified adjuvant therapy post-NAC; proposes MRI-quantified ITH as noninvasive prognostic tool
Hatano et al [26]	HER2 intratumoral genetic heterogeneity	Vacuum-assisted breast biopsy (VAB)	High HER2 heterogeneity was independently associated with significantly lower pCR rates and anti-HER2 resistance	HER2 ITH may function as a predictive biomarker of resistance to anti-HER2 neoadjuvant chemotherapy, enabling improved patient stratification and supporting personalized therapeutic strategies for HER2-positive breast cancer
Dai et al. [27]	Genetic HER2 ITH	Biopsy-to-surgery paired assessment	HER2 ITH may contribute to resistance by enabling coexistence of HER2-amplified and HER2-low resistant subclones that reduce sensitivity to anti-HER2 therapy	HER2 heterogeneity assessment may improve prediction of anti-HER2 NAC response and help identify patients who could benefit from intensified or alternative HER2-targeted treatment strategies
Tarantino et al. [25]	Genomic HER2 heterogeneity	Primary breast tumor tissue (FFPE)	HER2 heterogeneity was predictive of recurrence and may reflect intrinsic resistance; high HER2DX risk score correlated with worse outcomes	HER2DX may guide de-escalation: low-risk patients may safely omit Taxanes or benefit from shorter T-DM1 durations
Huang et al. [28]	Spatial radiomic and ecological ITH	Whole-tumor MRI	ITH spatial may reflect coexistence of treatment-sensitive and resistant tumor subregions, contributing to incomplete response and recurrence following NAC	MRI-based ITH quantification may serve as a noninvasive biomarker for predicting NAC response and recurrence risk, helping identify resistant tumors early and guide personalized treatment planning
Gandhi et al. [21]	Immune microenvironment heterogeneity	Pre-treatment biopsy + post-NAC surgical specimen	Chemo resistant tumors had a few cytotoxic CTLs and abundant α-SMA–positive stroma. This uneven immune and stromal composition likely hinders immune-driven tumor clearance and allows chemotherapy-resistant cells to persist.	CKM may enhance response to NAC by improving immune cell recruitment and remodeling the TME, potentially providing an alternative to immunotherapy or a de-escalation strategy for toxic chemotherapy
Lusby et al. [22]	Single-cell transcriptomics	Primary TNBC tumor tissue	Identified TFs and SPs drive expression of chemoresistance genes; intratumorally cell populations defined by these TFs are more chemo resistant	TFAP2C and SP1 knockdown enhances chemo efficacy; potential for TF-targeting therapies to overcome chemoresistance
Shi et al. [32]	Spatial radiomic heterogeneity	Whole-tumor MRI	Intratumoral ecological diversity may reflect coexistence of treatment-sensitive and resistant tumor subregions, contributing to variable response to NAC and incomplete pCR	MRI-derived ITH assessment may function as a noninvasive predictive biomarker for NAC response, enabling early identification of resistant tumors and supporting personalized treatment stratification and therapy optimization in breast cancer patients
Chitalia et al. [30]	Radiomics+ transcriptomics	Primary breast tumor tissue and DCE-MRI scans	ITH change patterns (radiomic) during NAC were predictive of treatment response and recurrence risk. Heterogeneity persistence or increase associated with poorer RFS.	Develop early imaging-based ITH change may allow risk stratification and personalized adaptation of NAC or adjuvant therapy. Suggests value of integrating MRI radiomics with genomics for decision-making.
Shaath et al. [23]	Transcriptomic ITH	Primary TNBC tumor tissue	MALAT1 overexpression contributes to NAC resistance through regulation of angiogenesis, oxidative phosphorylation, chromatin modification, and inflammatory pathways	Targeting MALAT1 may sensitize TNBC to chemotherapy and reduce resistant cell populations
Kim et al. [24]	Super-enhancer networks (epigenetic)	Pre-NAC biopsy and post-NAC residual tumor	Primed cells before treatment, expressing resistance-related genes in advance.	Potential to target resistance pathways (AKT1, EMT, hypoxia); early detection of resistant clones may improve patient stratification; potential for combination therapies to prevent resistance
Almendro et al. [31]	Genetic and phenotypic heterogeneity	Primary breast tumor tissue	Tumors with higher baseline genetic heterogeneity had poorer response (resistant), suggesting ITH contributes to therapeutic resistance	Measuring genetic diversity prior to treatment may help predict response to NAC. Topological/spatial assessment of ITH could refine therapeutic predictions

CTL= stands for Cytotoxic T Lymphocyte, α-SMA= Alpha–Smooth Muscle Actin, TFAP2C= Transcription Factor AP-2 gamma, SP1 = Specificity Protein 1, TF= Transcription Factor, SP= Super-enhancer Program, RFS= relapse -free survival.

### Quality assignment

The NOS scores for the included studies ranged from 6 to 9, indicating that all studies were of at least moderate methodological quality (Supplementary Table-1). Based on standard thresholds (≥ 7 = high quality; 5–6 = moderate quality), eight studies were considered high quality [21, 23, 24, 26–28, 30, 32], while four were assessed as moderate quality [22, 25, 29, 31].

## Discussion

This review highlights the evidence that ITH is associated with chemotherapy resistance in primary breast cancer. The twelve studies collectively use diverse methodologies including single-cell omics (DNA/RNA), epigenomics, radiomics (DCE-MRI), spatial in-situ profiling, and clinical trials to interrogate how ITH relates to chemotherapy response in primary breast cancer. Based on the results of selected studies, single-cell analyses showed that pre-existing genotypes can survive therapy (adaptive selection), while resistant expression programs are largely acquired during treatment, notably, a minority of pre-treatment cells appear primed, by expressing subsets of resistance-related genes in advance [24]. In addition, complementary work mapped super-enhancer–driven regulatory circuitry underlying chemoresistance and derived clinically relevant response signatures [22], while single-cell lncRNA profiling implicated MALAT1 as a mechanistic contributor to resistance [23]. Radiomics linked DCE-MRI heterogeneity phenotypes when combined with genomic assays to improved prediction of RFS after NAC [30], while Histopathological and spatial in-situ profiling provided direct visualization of ITH across treatment, showing stable genetic diversity but shifts in phenotypic composition, lower pre-treatment genetic diversity was associated with pCR [31]. MRI-based heterogeneity measured before and during NAC predicted pCR and early recurrence risk, with rising heterogeneity during therapy indicating worse outcomes [29]. In early TNBC, adding a systemic chemokine-modulatory regimen to standard NAC was feasible, showed an acceptable safety profile, and was associated with immune remodeling and a numerically favorable pCR rate [21].

### ITH as a pre-treatment risk indicator

The collected studies identified several distinct biological mechanisms through which ITH contributes to treatment resistance in primary breast cancer. First, in adaptive clonal selection, chemotherapy exerts selective pressure that favors the survival and expansion of pre-existing resistant subclones. This was evidenced by Kim et al.’s observation that certain tumor cell clones persist through treatment, indicating that therapy essentially prunes sensitive cells while allowing resistant clones to dominate [24].

Similar observations were reported in HER2-positive breast cancer, where both Tanei et al. [33]. and Dai et al. [27]. found that tumors with heterogeneous HER2 amplification patterns were significantly less likely to achieve pCR following anti-HER2 neoadjuvant therapy. These findings suggest that the coexistence of HER2-amplified and HER2-low subclonal populations may create a reservoir of intrinsically resistant cells before treatment begins, supporting the hypothesis that baseline heterogeneity may serve as an early predictor of treatment failure.

Second, dynamic changes in gene expression networks enable cancer cells to assume drug-tolerant states. Lusby et al. demonstrated that super-enhancer-driven TF circuits can reprogram TNBC cells, maintaining a pool of chemo resistant cells despite therapy [22]. Third, immune exclusion can contribute to resistance in heterogeneous tumors, as tumors with high spatial ITH often exhibit regions devoid of effective immune cell infiltration. Gandhi et al. found that areas with poor CTL penetration corresponded to lack of response, whereas interventions that increased T-cell infiltration (like CKM therapy) improved chemotherapy outcomes [21]. Fourth, epigenetic and post-transcriptional regulators such as long non-coding RNAs can drive resistance. Shaath et al. highlighted that upregulation of LncRNAs like MALAT1 in residual tumor cells is associated with therapy evasion, pointing to an additional layer of regulatory heterogeneity [23].

### ITH and treatment failure

Across subtypes, the reviewed evidence suggests that high or persistent ITH before or during therapy is associated with lower likelihood of pCR, greater residual disease (RD) burden, and poorer recurrence-related outcomes. For instance, Kim et al. [24] and Balko et al. [34] demonstrated that resistant clones pre-exist before therapy and are selectively enriched post-treatment. Shaath et al. [23] showed that lncRNA-mediated transcriptomic plasticity (e.g., MALAT1 overexpression) contributes to resistance in TNBC. Lusby et al. [22] highlighted how super-enhancer transcriptional circuits maintain cell states associated with resistance and progression.

These findings suggest that chemotherapy failure in some tumors may be driven by the survival and expansion of pre-existing resistant subclones rather than the acquisition of new mutations during treatment [24, 35].

Another mechanism may involve active maintenance of heterogeneous cellular states by the TME. Growth factors such as HGF and NRG1β have been shown to preserve diverse basal, luminal, and mesenchymal phenotypes during targeted therapy exposure, thereby promoting treatment resistance. Rather than simply selecting resistant clones, microenvironmental signaling appears to sustain phenotypic diversity throughout treatment. This observation supports the view that ITH is not solely a tumor-cell property but is also shaped by interactions between cancer cells and their surrounding microenvironment [36, 37].

Radiomics and MRI-derived heterogeneity metrics are emerging as promising, non-invasive predictors of response. Chitalia et al. [30] showed that early spatial-phenotypic shifts on MRI could predict recurrence and add predictive power to existing genomic classifiers like PAM50. Together, these studies suggest that imaging-derived measures of ITH could capture clinically relevant tumor diversity that is difficult to assess using conventional tissue sampling alone [38, 39]. Consistent with these findings, Huang et al. [28] showed that MRI-derived spatial heterogeneity independently predicted both pCR and RFS, while Shi et al. [32] demonstrated that ecological diversity-based MRI features improved prediction of pCR across multicenter cohorts. Together, these studies imply that imaging-derived measures of ITH may possibly capture clinically relevant tumor diversity that is difficult to assess using conventional tissue sampling alone. An important implication of ITH is that analyses based on small biopsy specimens, tissue microarrays, or limited omics samples may not fully capture the biological diversity present within a tumor. As a result, such approaches may underestimate clinically relevant resistant subclones or spatially distinct tumor ecosystems [15, 40]. This limitation highlights the importance of multimodal strategies that integrate imaging, spatial profiling, and molecular characterization to achieve a more comprehensive assessment of tumor heterogeneity [41].

TNBC studies [21–23] overwhelmingly demonstrated diverse ITH mechanisms including transcriptional noise, immune exclusion, and lncRNA involvement. In HER2-positive disease, multiple studies support the clinical relevance of genomic heterogeneity. Beyond the prognostic value of the HER2DX score reported by Tarantino et al. [25], both Hatano et al. [26]and Dai et al. [27] demonstrated that ITH in HER2 was associated with lower pCR rates and reduced responsiveness to anti-HER2 therapy.

Collectively, these findings suggest that heterogeneous HER2-amplified and HER2-low subclones may contribute to reduced sensitivity to targeted therapies [25]. The study by Almendro et al. [31] reminded us that phenotypic and genetic diversity do not always align, complicating treatment predictions. This heterogeneity underlines the need for multimodal profiling approaches, especially in tumors like TNBC where both the tumor and microenvironment exhibit profound plasticity [42].

Studies emphasized the importance of characterizing RD, which often harbors targetable pathways (e.g., PI3K, FGFR, JAK2) and may be overcome by immune modulation. Immunologic diversity in the TME may represent a potentially actionable dimension of heterogeneity [43]. Gandhi et al. [21] demonstrated that CKM increased CD8 + T-cell infiltration and improved pCR in TNBC. These insights support investigation of ITH-guided adaptive treatment strategies, in which therapy could be adjusted based on longitudinal heterogeneity signals. This remains a research question rather than a current clinical approach.

This review has several limitations. Although twelve studies met the inclusion criteria, they differed substantially in design, patient populations, ITH assessment methods, and outcome definitions, which precluded formal meta-analysis. Outcomes varied across studies and included pCR, RFS, OS, BCSS, iDFS, and RFI, while follow-up duration was inconsistent, limiting direct comparison and reducing the strength of conclusions regarding long-term outcomes such as late recurrence. Most studies were retrospective, single-center, and based on relatively small cohorts, increasing the risk of selection bias, limiting generalizability, and reducing power to detect smaller or subtype-specific effects. In addition, TNBC and HER2-positive tumors were overrepresented, whereas ER-positive/luminal disease was underrepresented, limiting confidence in the generalizability of the findings across all breast cancer subtypes. Another limitation is the lack of standardization in ITH measurement. The included studies used diverse methodologies, including radiomics, bulk and single-cell sequencing, transcriptomic profiling, multiplex immunohistochemistry, immune profiling, and spatial analyses, often with different metrics, thresholds, and workflows. As a result, direct quantitative comparison across studies remains difficult. Furthermore, tumor evolutionary dynamics cannot be directly assessed in patients achieving pCR, since no residual tumor tissue is available for post-treatment evaluation; therefore, analyses of pre- and post-treatment ITH are necessarily restricted to patients with residual disease and may be biased toward resistance-associated changes. Methodological caution is also needed in interpreting quality assessment scores, as some mechanistic or experimental studies do not fit neatly within standard cohort-based appraisal tools. Finally, the restriction to English-language, peer-reviewed publications introduces the possibility of language and publication bias, and the rapidly evolving nature of the field means that newly relevant studies may have appeared since the original search.

Despite these limitations, the findings were consistent in supporting an association between higher ITH and poorer treatment response. Future large-scale, multicenter prospective studies using standardized and harmonized approaches to heterogeneity assessment, together with long-term outcome evaluation, are needed to validate these findings, facilitate the clinical translation of ITH assessment, and support its integration into treatment decision-making.

## Conclusion

ITH appears to be an important contributor to chemotherapy resistance in primary breast cancer, particularly in aggressive subtypes such as TNBC. This review study showed that, higher or persistent molecular, genomic, and spatial heterogeneity was generally associated with poorer treatment response and less favorable clinical outcomes, although the strength and consistency of these associations varied by study design, tumor subtype, and analytical approach. Taken together, the current evidence might support a link between ITH and treatment failure, while also underscoring the methodological limitations and heterogeneity of the existing literature.

Beyond its prognostic relevance, several studies suggest that ITH is a dynamic rather than entirely fixed feature of tumors. Early multimodal approaches integrating radiogenomic, immunogenomic, and microenvironment-related measures are promising, but remain exploratory. Further prospective and longitudinal studies combining advanced imaging, single-cell and spatial omics, and immune profiling are needed to determine whether heterogeneity-guided approaches can improve prediction of resistance and help inform treatment selection. Clinical translation will require standardized methods for measuring ITH and carefully designed studies to establish whether and under what conditions ITH-informed approaches can improve patient outcomes.

## Data Availability

No datasets were generated or analysed during the current study.

## Abbreviations

AC: Adjuvant chemotherapy
α-SMA: Alpha-smooth muscle actin
BCSS: Breast cancer-specific survival
CFU: Colony-forming unit
CI: Confidence interval
CKM: Cytokine-kinase mediated
CNV: Copy number variation
CTL: Cytotoxic T lymphocyte
DCE-MRI: Dynamic contrast-enhanced magnetic resonance imaging
DFS: Disease-free survival
EMT: Epithelial-mesenchymal transition
ER: Estrogen receptor
HER2: Human epidermal growth factor receptor 2
HR: Hazard ratio
ICGS: Iterative clustering and guide-gene selection
IHC: Immunohistochemistry
IPA: Ingenuity pathway analysis
ITH: Intratumor heterogeneity
lncRNA: Long non-coding RNA
MALAT1: Metastasis-associated lung adenocarcinoma transcript 1
MICSSS: Multiplexed immunohistochemistry with spatial single-cell segmentation
NAC: Neoadjuvant chemotherapy
NOS: Newcastle–Ottawa Scale
OR: Odds ratio
OS: Overall survival
pCR: Pathologic complete response
RD: Residual disease
RFI: Recurrence-free interval
RFS: Relapse-free survival
scRNA-seq: Single-cell RNA sequencing
SE: Super-enhancer
SP1: Specificity protein 1
TF: Transcription factor
TFAP2C: Transcription factor AP-2 gamma
TME: Tumor microenvironment
TNBC: Triple-negative breast cancer
